# Generative LLMs for educational recommendation

**DOI:** 10.3389/frai.2026.1827553

**Published:** 2026-08-19

**Authors:** Wang Yun, Bekarystankyzy Akbayan, Kassenkhan Aray, Tolegenov Azamat

**Affiliations:** 1 Institute of Automation and Information Technologies, Satbayev University, Almaty, Kazakhstan; 2 Narxoz University, Almaty, Kazakhstan; 3 Satbayev University, Almaty, Kazakhstan

**Keywords:** educational recommendation, explainable recommendation, large language models, learning analytics, online learning

## Abstract

Online learning platforms contain large question banks, yet learners often lack clear guidance on what to practice next and why a particular item is appropriate. Existing educational recommender systems can optimize learning paths, but their decisions are often difficult for learners to interpret. Large language models (LLMs) can generate natural-language explanations, but unconstrained generation may introduce hallucinated learner histories or unsupported pedagogical claims, which is problematic in educational scenarios. To address this issue, we propose an evidence-grounded closed-loop educational recommendation framework that combines lightweight learner-state tracking with controllable generative explanations. In this system, a deterministic ranking module selects candidate exercises using pedagogical signals such as mastery gaps and learning efficiency. The LLM is restricted to retrieved learner logs and instructional metadata and must provide explanations with explicit evidence references. We evaluate the framework through system benchmarks, simulation-based ranking comparisons, and manual assessment of explanation quality. The results suggest that the framework is feasible for online deployment, that the hierarchical efficiency strategy improves skill coverage and learner differentiation in simulation, and that evidence-grounded prompts improve explanation usefulness while reducing observed fabricated-history cases in sampled outputs. Overall, the study shows that separating deterministic recommendation from evidence-supported explanation can improve the credibility and practical utility of LLM-assisted educational recommendation.

## 1 Introduction

The growth of MOOCs and intelligent tutoring platforms has made a lot of educational content available. However, personalized guidance is still limited. Learners are often confronted with thousands of exercises under each topic, as well as a variety of learning resources. Without effective support to choose the right exercises and the reasons for the recommendations, learners may practice inefficiently, over-practice simple items or lose motivation because they are not clear on their progress.

Unlike entertainment recommendation systems, educational recommendation objectives are different. In education, recommendations are focused on building an effective learning path, determining the concepts that influence the practice of the learners, the best order of exercises, and proposing learning methods. Consequently, the interpretability and reliability of recommendations is not a measure of “how good” they are; it is central to the overall effectiveness of the system.

### 1.1 The explainability–reliability tension

Existing educational recommendation systems often try to identify knowledge gaps of learners and push content that targets weak skills in order to maximize “learning gains.” However, if the learners do not know why certain exercises are recommended or how to act on them, it is not enough to present “appropriate” questions. Explanations are important, because they influence the metacognition of learners, that is, the processes by which learners plan, monitor, and modify their learning behaviors.

Traditional recommender systems can recommend exercises based on latent-factor models or other predictive signals, but they often provide limited semantically meaningful explanations for their recommendations. This limitation is especially consequential in education: whereas a poor movie recommendation may simply waste time, an unclear or mismatched educational recommendation can interfere with learners’ metacognitive strategies and learning outcomes. Explainable recommender systems have therefore been studied extensively as a way to make recommendations more transparent and understandable, with surveys emphasizing the need to align recommendations with user behaviors and learning goals (Zhang and Chen, 2020).

The advent of generative large language models (LLMs) holds the promise of bridging this gap by acting as always-available tutors. However, there is a new reliability paradox when it comes to deploying generative LLMs: models that are fluent enough to act like tutors are also likely to generate plausible but factually incorrect hallucinations (Ji et al., 2023). In high-stakes educational situations, even a single hallucinated formula or wrong strategy can cause the learner to lose trust. As a result, the central research question in this era becomes:

Thus, the central research question in this era evolves from “How to recommend accurately?” to “How to effectively harness the generative power of LLMs for explanation without compromising pedagogical rigor?”

Existing research is missing mechanisms for LLMs to explicitly reference user behaviors, such as “you encountered difficulties in question X” or “weak recursive ability.” To overcome this shortcoming, our work presents a reference-forced prompting strategy, which extends beyond purely semantic relationships and is structured and verifiable. Explanations generated using this strategy are evidence-based, making reference to specific IDs from learner logs and teaching metadata, and thus providing the possibility of evidence-based evaluation of the system.

### 1.2 Our approach: evidence-grounded generative recommendation

We argue that a practical educational recommendation system (edu-recommender) should be based on a strict division of labor.

First, ranking should be determined based on interpretable signals and instructional metadata to select appropriate content.

Second, generative models need to be constrained by verifiable evidence in order to convert recommendations into actionable explanations.

To this end, we propose an evidence-based closed-loop framework combining lightweight mastery tracking with a citation-mandatory explanation module. In this design, LLMs are not used as knowledge bases, but instead as reasoning and communication interfaces over retrieved evidence. This approach clearly separates knowledge sources, for example, indexed databases and learner logs, from the interface layer, which is represented by the LLMs.

### 1.3 Contributions

This work makes the following contributions:

After each learning attempt, the closed-loop framework updates the state of the learner and efficiently recalculates recommendations.

Guided by evidence and operating under a citation-mandatory prompt, LLMs explicitly include evidence IDs for each explanation point.

The methodological contribution is the controlled integration of deterministic educational ranking, structured evidence construction, citation-enforced prompting, and auditable explanation generation within a closed-loop learner-state system. The contribution lies in the controllability and auditability of LLM-generated explanations, not in the ranking formula itself, which intentionally uses lightweight and interpretable heuristics.

Empirical insights from this framework include: (i) evidence grounding reduces observed fabricated-history risk in sampled explanations by making missing data explicit, and (ii) the hierarchical efficiency framework enhances early differentiation by adding learning speed, helping distinguish fast learners from learners who may need additional support.

## 2 Related work

### 2.1 Educational recommendation and learner modeling

Educational recommendation systems include learning path recommendations, curriculum sequencing, and exercise recommendations, and are guided by learner models. Knowledge tracing (KT) and its variants on attempt sequences are usually used to estimate skill mastery (Corbett and Anderson, 1994), on which a basis for recommending items that are associated with unmastered concepts. For a taxonomy of KT models, variants and applications, see Shen et al. (2024). Beyond traditional classroom-based KT, deep learning (DL) approaches, such as deep knowledge tracing (DKT), learn representations from interaction sequences to allow large-scale modeling of knowledge evolution (Piech et al., 2015). More recently, KT has been reformulated as a sequence modeling problem, where learning traces are treated as language-like data streams (Zhan et al., 2024). Additionally, by considering teaching as a strategy optimization problem, reinforcement learning-based approaches can take exploration and long-term rewards into account. However, the practical deployment of these methods often encounters issues with data availability and model stability.

### 2.2 The shift to generative recommendation

Traditionally, recommender systems have been discriminative: they rank existing items. More recently, the field has moved toward generative recommendation (Wang et al., 2025), in which models can also produce content such as explanations, summaries, user profiles, or learning materials. Surveys often distinguish roles such as “LLMs as rankers” and “LLMs as explainers” (Wu et al., 2024), and describe LLM-enhanced recommender systems as modular architectures in which LLMs support reasoning, interaction, or generation (Liu et al., 2025). The “LLMs as rankers” paradigm is promising, especially when LLMs are tuned for recommendation objectives, but latency and controllability can be problematic in large-scale MOOCs that require fast responses. Our work belongs to the “LLMs as explainers” paradigm, but applies stricter constraints: the LLM is not treated as a knowledge base or ranking model, but as a natural-language explanation layer operating over retrieved learner and item evidence. This design separates knowledge sources, such as indexed course data and learner logs, from the generative interface layer.

### 2.3 The “grounding” problem in education

Retrieval-augmented generation (RAG) conditions language generation on retrieved external evidence to support knowledge-intensive tasks and reduce hallucination risk (Lewis et al., 2020). However, conventional RAG can retrieve loosely related text and may not enforce faithful attribution at the level of individual claims. Recent work has therefore examined answer attribution in RAG systems, including model-internals-based methods such as MIRAGE (Li et al., 2024; Gao et al., 2024). Peer-reviewed RAG evaluation studies further show that evaluation should consider retrieval relevance, generated-answer faithfulness, citation quality, latency, and robustness rather than treating generation quality as a single end-stage score (Brown et al., 2025). Retrieval-focused evaluation work also shows that retrieval-stage quality and downstream generation quality do not always align, which motivates pipeline-level evaluation of evidence use (Salemi and Zamani, 2024). In education, grounding should be stricter: an explanation must be consistent with the learner’s interaction history, current learner-state estimate, and the relevant pedagogical procedure.

Table 1 summarizes the main research streams discussed above and clarifies how the proposed framework differs from existing approaches.

**Table 1:** Comparative overview of related approaches and the proposed framework.

Research stream	Typical focus	Limitation for this study	How this work differs
Educational recommendation and KT (Corbett and Anderson, 1994; Shen et al., 2024; Piech et al., 2015; Zhan et al., 2024)	Estimate learner mastery and recommend exercises or learning paths.	Often prioritizes ranking or mastery prediction, with limited natural-language explanation.	Keeps ranking deterministic but adds evidence-grounded explanations for pedagogical scaffolding.
Explainable recommendation (Zhang and Chen, 2020)	Improve transparency and user understanding of recommendations.	General XRec explanations may be preference-oriented and not tied to learner-state evidence.	Requires explanation claims to be supported by learner logs, mastery estimates, and item metadata.
LLM-enhanced recommendation (Wang et al., 2025; Wu et al., 2024; Liu et al., 2025)	Use LLMs as rankers, explainers, or generative components.	LLM-as-ranker designs can be costly and may introduce uncontrolled generation.	Uses the LLM only as a constrained explanation layer, not as the source of ranking decisions.
RAG and grounded generation (Lewis et al., 2020; Li et al., 2024; Brown et al., 2025; Salemi and Zamani, 2024)	Condition generation on retrieved evidence to improve attribution and faithfulness.	Standard RAG may retrieve loosely related text and may not enforce claim-level learner consistency.	Uses structured evidence IDs and citation rules to support auditability in educational recommendations.
Proposed framework	Closed-loop educational recommendation with citation-enforced explanations.	Current validation remains simulation-based and requires stronger real-learner evaluation.	Combines mastery-aware ranking, evidence construction, citation enforcement, and groundedness metrics.

#### 2.3.1 Positioning relative to existing paradigms

The proposed framework connects three research traditions but differs from each of them in scope and control mechanism. Compared with explainable recommender systems, it keeps the recommendation score deterministic while requiring the generated explanation to be grounded in learner-state and item evidence. Compared with standard RAG, it does not retrieve open-ended text passages for general question answering; instead, it constructs a structured evidence bundle from learner logs, mastery estimates, problem metadata, and skill tags. Compared with grounded LLM architectures, it uses the LLM only after the ranking decision has been made, so generation cannot change the recommended item and can only explain it using labeled evidence. This positioning is important for educational recommendation because unsupported personalization can misrepresent a learner’s history or state.

## 3 Theoretical framework: cognitive trust and scaffolding

Interpretation of incentive evidence based on two educational theories.

### 3.1 Instructional scaffolding

Inspired by Vygotsky’s theory, instructional scaffolding argues that learners grow by using a support system that bridges from one’s current abilities to one’s potential abilities. In recommendation systems, engines often calculate abstract signals, like “skill gaps,” but learners need guidance on how to turn these signals into actionable steps.

In our framework, the explanation module is a dynamic scaffolding. It translates the quantitative results of the recommendation engine, such as mastery gaps, recent error patterns, and other learner-state indicators into natural language, concise suggestions. These explanations help to explain the choice of a particular question and offer guidance on how learners should approach the question.

### 3.2 Trust calibration

Within the educational artificial intelligence, students need to tune their trust, not to blindly trust AI suggestions and not to neglect helpful advice. A proper trust calibration must have a clear foundation: learners should be aware of the logic behind the suggestions of the system. As an illustration, one can give explanations that cite the obligatory evidence, like mastery scores, and this will give the learners the tools to check the logic of the system and create the proper trust. Additionally, this is the principle behind our fundamental philosophy of design: explanations must be verifiable and not just persuasive.

## 4 Problem setting and design principles

### 4.1 Instructional scaffolding task definition

For a learner *u* enrolled in course *C*, let *P*_*C* denote the set of candidate problems available for recommendation. The system uses the learner state, recent interaction history, and problem metadata to select a top-*k* list.

The output is *R*(*u*, *C*) = {(*p*₁, exp.₁), …, (*pₖ*, exp*ₖ*)}, where *pᵢ* denotes the *i*-th recommended problem and exp*ᵢ* denotes its optional evidence-grounded explanation.

Each explanation should specify: (1) the skill or skills targeted by the problem, (2) why the problem is recommended given the learner state, and (3) how the learner should approach the problem.

Specific factual claims in expᵢ must be supported by evidence IDs from the evidence bundle *E*(*u*, *pᵢ*).

When relevant evidence is unavailable, the explanation must acknowledge the missing information rather than infer unsupported learner history.

### 4.2 Design principles

The system separates responsibilities: it decides on the order in which recommended content should be chosen, while LLMs use evidence to explain the reason behind each recommendation.

The interpretation of evidence in this structured framework does not depend on external knowledge.

We construct an explainable format that is auditable and contains clear references that can be used for verification.

With regard to deployability, LLM calls are on-demand with timeout and fallback mechanisms and the core recommendation computations are efficient.

## 5 Methodology

### 5.1 Closed-loop learner state tracking

For each skill *s*, a mastery estimate *p_u_(s)* is maintained within the interval (0, 1). When presenting a question associated with a skill set *S(p)* the system updates the mastery estimates using interpretable online rules.

- If correct: *p′ = min(0.99, p + α(1 − p))*.
- If incorrect: *p′ = max(0.01, p − βp)*.

The parameters alpha and beta serve as hyperparameters. In the reported simulation, alpha = 0.15 for a correct response and beta = 0.10 for an incorrect response; a threshold tau = 0.80 is used to determine when a skill is considered mastered.

We use this lightweight knowledge-tracing-style update because it is stable, interpretable, and suitable for online recommendation. In the simulation study, we compare it with a Bayesian Knowledge Tracing (BKT) baseline to contextualize its behavior.

### 5.2 Multi-objective ranking: gain and efficiency

For each skill *s*, the expected gain is defined as gain(*s*) = 1 − *p*_*u*(*s*), where *p*_*u*(*s*) is the current mastery estimate of learner *u* for skill *s*.

We consider an efficiency signal derived from learner attempts. Let *k*_*u*(*s*) denote the number of attempts made by learner *u* on skill *s*. A simple attempt-based efficiency proxy is eff(*s*) = 1/(1 + *k*_*u*(*s*)).

To address the late-binding problem, where attempt counts alone provide weak early differentiation, we adopt a hierarchical efficiency framework.

Layer 1 (Mastered): If *attempts-to-mastery* exists, *eff = 1/(1 + attempts-to-mastery).*

Layer 2 (Learning History ≥ 3): If mastery-history length ≥ 3, compute learning velocity *v* = (mastery[last] − mastery[first])/(len − 1), then set eff(*s*) = sigmoid(15*v*), bounded below by 0.3 and above by 1.0.

Layer 3 (cold start): If total attempts = 0, *eff = 1.0* (exploration bonus).

Layer 4 (Early Learning): *eff = 1/(1 + current-attempts)*.

For a candidate problem *p* with skills *S(p)*, the score is:

*Score(p) = ∑_s∈S(p)_ (w_g_·gain(s) + w_e_·eff(s))*.

We compare strategies such as gain-only (*w_e_ = 0*) and gain + efficiency with different definitions of *eff(s)*.

#### 5.2.1 Worked example of score computation

The following example illustrates how the equations are applied. It is not an additional experiment or a real learner record; it uses the default implementation parameters reported in Table 2.

**Table 2:** Key hyperparameters and evaluation settings.

Setting	Value used	Source/role
Top-*k* recommendation size	*k* = 10	Number of recommended problems per step.
Candidate cap	max_candidates = 2000	Maximum unseen candidate problems scored at each recommendation step.
Episode length	40 steps	Number of simulated learning interactions per synthetic learner.
Seeds	50 seeds per strategy per course	Produces 500 simulated learners across the reported course set.
Initial missing mastery	0.20	Fallback mastery estimate when no skill history exists.
Mastery threshold	0.80	Threshold for considering a skill mastered.
Full/full-v2 weights	gain = 0.6, efficiency = 0.3	Ranking weights used by the simulation scoring function.
BKT transition	*P*(*T*) = 0.15	Learning probability in the BKT baseline.
BKT slip/guess	*P*(*S*) = 0.10, *P*(*G*) = 0.20	Observation-noise parameters in the BKT baseline.
Simulated response model	response_slope = 6.0, learn_rate_correct = 0.10	Synthetic learner dynamics used to generate correctness and mastery updates.
Confidence intervals	1,000 bootstrap resamples, 95% CI	Used for the summary statistics in Table 6.
Lightweight mastery update	Alpha = 0.15, beta = 0.10	Online mastery update used by the lightweight recommender simulation.

Step 1: Mastery update. Suppose learner u has an estimated mastery *p*_*u*(*s*) = 0.40 for skill s and answers an item correctly. Using the lightweight update rule with alpha = 0.15, the updated mastery is *p*_new = min(0.99, 0.40 + 0.15(1–0.40)) = min(0.99, 0.49) = 0.49. If the response had been incorrect, the corresponding update with beta = 0.10 would be *p*_new = max(0.01, 0.40–0.10*0.40) = 0.36.

Step 2: Gain. Before the update, the gain proxy is gain(s) = 1−*p*_*u*(*s*) = 1–0.40 = 0.60. After a correct response, the gain becomes 1–0.49 = 0.51, showing that the same skill becomes less urgent once mastery increases.

Step 3: Efficiency. If the learner has not yet mastered the skill and has current_attempts = 2, the full-v2 early efficiency signal is eff(*s*) = 1/(1 + 2) = 0.333. If the skill has already reached mastery after a attempts-to-mastery value of 3, the mastered-layer efficiency would be eff(*s*) = 1/(1 + 3) = 0.25.

Step 4: Final score. For a candidate problem *p* involving only skill *s*, using *w*_*g* = 0.6 and *w*_*e* = 0.3, the pre-update full-v2 score is Score(*p*) = 0.6*0.60 + 0.3*0.333 = 0.460. After the correct response, if the same efficiency value were used for illustration, the score would become 0.6*0.51 + 0.3*0.333 = 0.406. This example shows how mastery gaps and efficiency jointly determine the ranking score.

### 5.3 Evidence construction: what the LLM is allowed to use

Given a candidate problem *p* and learner *u*, the system constructs an evidence bundle *E*(*p*, *u*) from the following sources.

1. Problem evidence: content/metadata/skill tags,
2. Mastery evidence: per-skill mastery summary for *S(p)*,
3. Attempt evidence: recent attempts filtered by skill overlap with *S(p)*,

Optional video evidence is for aligned video cues.

This evidence bundle is the only factual source that the LLM is allowed to use when generating an explanation.

### 5.4 Citation-enforced prompting strategy

To avoid mixing manuscript prose with model-directed instructions, we separate the citation-enforced prompt into explicit components. The prompt template below summarizes the citation-enforced prompt used by the explanation module (see Table 3).

**Table 3:** Prompt template. Citation-enforced prompt template.

Prompt component	Purpose	Instruction in the template
Role and task	Define the LLM as an explanation layer, not a ranker.	Explain why the selected problem is recommended and how the learner should approach it.
Evidence injection	Provide auditable context.	Use only labeled evidence blocks such as [E1], [E2], and [E3].
Citation rule	Support structural verification.	Every specific factual claim must cite at least one valid evidence ID, for example [#E2].
Missing-data rule	Reduce fabricated learner histories.	If evidence is missing, explicitly state that the system has no evidence rather than inferring prior attempts or assignments.
Output format	Keep explanations actionable and comparable.	Return concise bullet points covering target skill, recommendation reason, and study strategy.
Prohibited behavior	Prevent unsupported personalization.	Do not invent learner scores, attempts, assignments, preferences, or private histories not present in the evidence bundle.

During generation, the model is instructed to use only the provided evidence bundle and to attach evidence IDs to specific factual claims (Wei et al., 2022).

This prompt design operationalizes evidence conditionalization at the system level: the LLM acts as a natural-language explanation layer over structured evidence rather than as an unconstrained knowledge source.

### 5.5 Strict groundedness: formal definition and metrics

We define groundedness using strict criteria so that explanations are evaluated according to citation coverage, citation validity, and claim-level support rather than only semantic relevance.

An explanation consists of *K* key points, denoted as *X* = {*x*1, …, *xK*}, where each key point *xk* is linked to a set of cited evidence IDs *C*(*xk*).

Structural groundedness (automatically checkable).

- Citation coverage: *Cov(X) = (1/K)∑k = 1…K 1[|C(xk)| > 0].*
- Citation validity: *Val(X) = (1/K)∑k = 1…K 1[∀e ∈C(xk), e ∈E].*

An explanation is structurally grounded when *Cov(X)* = 1 and *Val(X)* = 1.

Semantic groundedness (claim support).

For each bullet point *xk*, we assess whether its claim is supported by the cited evidence and assign a score as follows.

- 2 = fully supported.
- 1 = partially supported (the general strategy is supported, but key factual details are missing).
- 0 = unsupported, contradicted, or containing unverifiable factual claims.

After scoring, normalize each point as *g(x_k_) = score(x_k_)/2*, and then *SemG(X) = (1/K)∑_k = 1_…K g(x_k_)*.

The requirements targeted by this strict definition are that explanations must be verifiable based on the learner’s current state and historical interactions.

#### 5.5.1 Operationalization of hallucination risk

In this study, hallucination risk is operationalized narrowly for educational recommendation rather than treated as a general open-domain factuality benchmark. We focus on whether the explanation invents learner-specific facts that are absent from the evidence bundle, such as prior attempts, assignments, scores, preferences, or mastery histories. The reported groundedness metrics therefore evaluate evidence traceability and claim support. They do not constitute a standardized hallucination benchmark; instead, they support the narrower claim that evidence-grounded prompting reduces observed unsupported learner-history claims in the sampled recommendation explanations (see Table 4).

**Table 4:** Groundedness and hallucination-risk evaluation signals.

Evaluation signal	What it detects	Interpretation and limitation
Structural groundedness	Whether every factual claim includes a valid evidence ID from the supplied bundle.	Automatically checks citation coverage and citation validity; it does not by itself prove semantic truth.
Semantic groundedness	Whether cited evidence fully, partially, or does not support each key claim.	Manual claim-support assessment on sampled outputs; useful for diagnosis but not a large-scale hallucination benchmark.
Fabricated-history check	Whether the explanation invents prior attempts, assignments, scores, preferences, or learner histories not present in evidence.	Directly targets the education-specific hallucination risk discussed in this paper; larger standardized evaluation remains future work.
Usefulness	Whether the explanation helps a learner understand and act on the recommendation.	Measures perceived pedagogical value; it is not a hallucination detector.

## 6 System framework (condensed)

This system adopts an architecture that separates offline indexing from online recommendation and explanation generation.

The offline index is built from source data and includes artifacts such as problem-skill associations and optional content-similarity measures.

During online operation, the system provides application programming interfaces (APIs) for recommendation, attempt submission, mastery updating, and on-demand explanation generation.

To keep the manuscript journal-focused, we provide the structure of the system at the framework level, without including many low-level details such as table fields.

**Figure 1:**
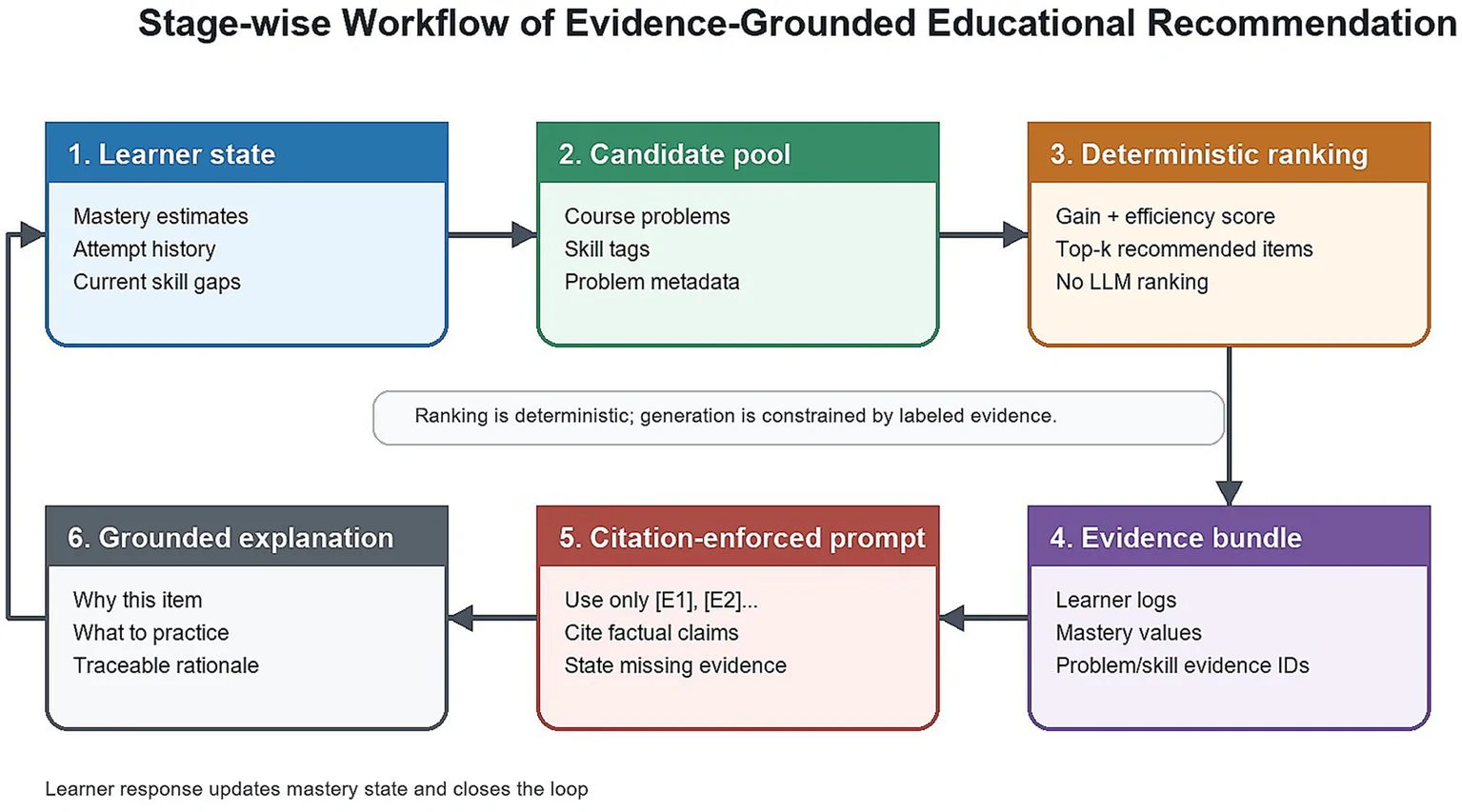
Stage-wise workflow of the proposed evidence-grounded educational recommendation framework. The diagram shows the closed loop from learner state and candidate ranking to evidence construction, citation-enforced prompting, grounded explanation, and learner-state updating.

Figure 1 provides a stage-wise overview of the proposed approach, showing how deterministic recommendation, evidence construction, citation-enforced prompting, and learner-state updating are connected in the closed loop.

## 7 Quantitative experiments

### 7.1 Experimental setup

We evaluate the proposed framework in two complementary ways: a simulation-based offline ranking study and a first-author-reviewed explanation-quality evaluation. The ranking study uses synthetic learner interactions to compare recommendation strategies under controlled assumptions. The explanation study examines whether evidence-grounded prompting makes generated explanations more traceable and useful across 50 recommendation instances and 100 generated explanation outputs, but it should still be read as a controlled annotation study rather than a full-scale classroom user study.

#### 7.1.1 Scope of validation

No longitudinal real learner interaction dataset was available for the present revision. Therefore, the ranking results should be interpreted as simulation-based offline evidence rather than as causal evidence of learning gains for real students. The simulation tests internal consistency, scalability, and comparative behavior of ranking strategies under explicit learner-response assumptions. Real-learner validation with platform logs, delayed post-test outcomes, and classroom deployment data remains necessary future work.

### 7.2 Strategy evaluation results

Within this validation scope, we evaluated ranking strategies using representative simulations.

- *N* = 500 synthetic learners over 40-step learning episodes.
- Skill mastery evolves via probabilistic learning dynamics (response_slope = 6.0, learn_rate_correct = 0.10).
- Each interaction updates mastery estimates, which feed subsequent recommendations.

We compared six ranking strategies: random, gain-only, full (gain plus efficiency), full-v2 (early efficiency signal), BKT, and IRT-style difficulty matching. Tables 2, 5 define these baselines and the key hyperparameters used in the simulation reported in Table 6.

**Table 5:** Baseline definitions used in the simulation study.

Strategy	Ranking rule	Purpose / note
Random	Uniformly shuffles unseen candidates and selects the top-*k* items.	Lower-bound baseline for ranking.
Gain-only	Scores each candidate by the mean mastery gap over its skills: mean(1 − *p*_mastery(*s*)).	Tests whether targeting weak skills alone is sufficient.
Full	Combines mean gain and attempt-to-mastery efficiency: 0.6 * gain + 0.3 * efficiency. If a skill has not been mastered, efficiency defaults to 0.5.	Main gain-plus-efficiency baseline.
Full-v2	Uses the same gain weight but replaces the missing mastery-efficiency default with an early signal: efficiency = 1/(1 + current_attempts) for not-yet-mastered skills.	Adds an early efficiency signal for cold-start differentiation in the simulation.
BKT	Updates estimated mastery with a two-state Bayesian Knowledge Tracing update using *P*(*T*) = 0.15, *P*(*S*) = 0.10, and *P*(*G*) = 0.20, then ranks using the resulting mastery estimates.	Compares the lightweight update against a classical KT-style baseline.
IRT-style	Ranks by difficulty matching: ability is the mean estimated mastery of the item skills, pseudo-difficulty is deterministically derived from the problem-id hash in [0,1], and score = 1 − |ability − difficulty|.	A simplified difficulty-matching heuristic, not a calibrated probabilistic IRT model.

**Table 6:** Simulation results: strategy comparison (mean ± 95% CI).

Strategy	*N*	Δ mastery	Post-test	Skill coverage	Skill overlap	Latency (s)
Random	500	0.086 ± 0.006	0.635 ± 0.016	9.7 ± 0.5	0.361 ± 0.025	0.0001
Gain-only	500	0.100 ± 0.006	0.483 ± 0.016	11.9 ± 0.4	0.199 ± 0.023	0.0002
Full (gain + eff)	500	0.100 ± 0.006	0.483 ± 0.016	11.9 ± 0.4	0.199 ± 0.023	0.0003
Full-v2 (layered)	500	0.100 ± 0.006	0.454 ± 0.018	21.0 ± 1.1	0.148 ± 0.025	0.0003
BKT	500	0.100 ± 0.006	0.482 ± 0.016	11.9 ± 0.4	0.199 ± 0.023	0.0003
IRT	500	0.075 ± 0.006	0.642 ± 0.014	5.9 ± 0.4	0.604 ± 0.025	0.0026

**Figure 2:**
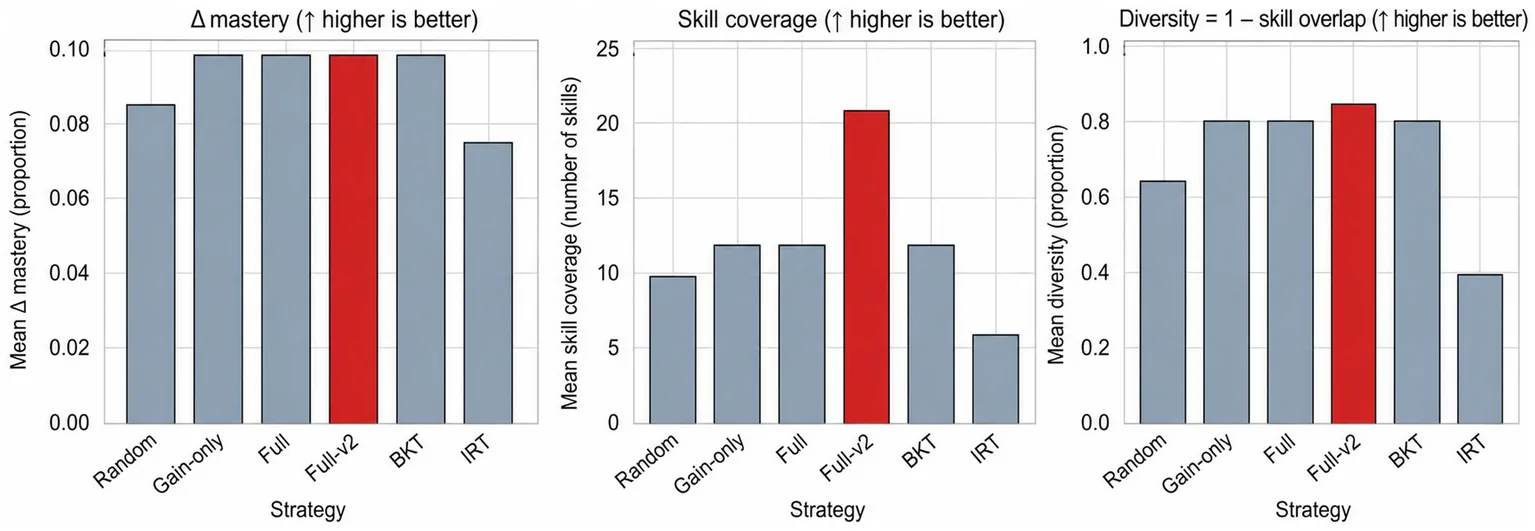
Comparison of full-v2 multi-objective performance with baseline strategies.

Figure 2, Table 6 summarize the simulation results. Full-v2 primarily improves the diversity-oriented objectives: it covers more skills and yields lower skill overlap than the other strategies. Its delta-mastery value is comparable to gain-only rather than clearly superior, so the result should be read as a multi-objective trade-off under simulation assumptions rather than as evidence of superior real-world learning outcomes.

Key findings: (1) In the simulation, gain-only achieved higher delta mastery than random under a two-sided bootstrap comparison with 1,000 resamples (*p* = 0.002; mean and 95% CI are reported in Table 6). (2) Full-v2 had higher skill coverage (21.0) and lower skill overlap (0.148), suggesting broader and less redundant practice; however, its delta mastery was not significantly different from gain-only in this simulation (*p* = 0.944). (3) The hierarchical efficiency framework is intended to differentiate learners after 3–5 attempts by assigning higher efficiency to faster learning trajectories and lower efficiency to slower trajectories.

#### 7.2.1 Effect size interpretation

For the gain-only strategy versus random recommendation on delta mastery, the mean difference is approximately 0.0138 (0.0996 vs. 0.0859). Because the stored evaluation file reports bootstrap confidence intervals rather than raw per-episode outcomes, Cohen’s *d* was approximated by recovering the standard deviation of each group from the reported 95% CI and *n* = 500. This gives Cohen’s *d* of approximately 0.22, a small effect by conventional standards. Under the simulation’s mastery ceiling and short interaction horizon, this should be interpreted as a modest efficiency gain rather than a large learning-outcome effect.

### 7.3 Explanation quality results

We evaluated 100 explanation outputs from 50 sampled recommendation instances. For each instance, we compared two explanation settings: one unconstrained explanation without evidence grounding and one evidence-grounded explanation generated with the structured evidence bundle. This yielded 50 outputs per condition. The original 100-output generation used the evaluation-script settings temperature = 0.3 and max_tokens = 512. The final annotation recorded truncation in 9/100 outputs overall: 8/50 without evidence and 1/50 with evidence. The first author reviewed and finalized the annotations for all 100 outputs.

#### 7.3.1 Annotation protocol

The 100 outputs were assessed by a single evaluator, the first author, with domain expertise in educational technology. The evaluator has master’s-level training in educational technology and educational recommender systems and was familiar with learner-state modeling, recommendation explanations, and the course-domain context used in this study. The evaluation considered usefulness, structural groundedness, and strict semantic support against the supplied evidence bundle. Inter-rater reliability was not computed because the current study used one evaluator; this is a limitation rather than a reliability claim. Future work will use multiple independent annotators and report formal agreement measures such as Krippendorff’s alpha.

#### 7.3.2 Annotation boundary

Structural groundedness and semantic groundedness were intentionally separated. Structural groundedness required an explicit traceable reference to an evidence element, such as a skill ID, problem ID, evidence ID, or named evidence field. Semantic groundedness required the main factual claims to be verifiable from the evidence bundle. Therefore, a numeric paraphrase such as “20% mastery” could be counted as semantically grounded when it matched *p*_mastery = 0.20, even if it did not satisfy the stricter structural citation criterion. Earlier small-batch strict checks were used as diagnostic pilots; Table 7 reports the final first-author-reviewed 100-output annotation under this clarified two-level criterion.

- Unconstrained LLM: instructed to explain without an evidence bundle.
- Evidence-grounded (ours): prompted with an evidence bundle and citation enforcement.

**Table 7:** Explanation quality evaluation on 100 outputs from 50 recommendation instances.

Method	Structural groundedness/citation traceability (%)	Strict semantic groundedness (%)	Usefulness (mean ± SD, 1–5)
Unconstrained LLM	0.0	0.0	2.84 ± 0.37
Evidence-grounded (ours)	90.0	68.0	3.74 ± 0.44

##### 7.3.2.1 Metrics

Usefulness (1–5 points) measures whether an explanation helps learners understand the recommendation and take appropriate action. Structural groundedness measures whether each explanation contains valid evidence citations. Because unconstrained explanations are generated without evidence IDs, their structural groundedness is 0 by design and should be interpreted as an evidence-traceability measure rather than a standalone hallucination rate. Semantic groundedness evaluates whether the cited evidence actually supports each key claim. We treat semantic groundedness as a stricter diagnostic measure because citation presence alone does not guarantee full claim support.

#### 7.3.3 Interpretation of strict groundedness

The strict semantic-groundedness checks indicate that citation enforcement improves traceability but does not fully solve unsupported generalization. Some evidence-grounded explanations may still introduce study-strategy claims that go beyond the supplied evidence. This finding motivates future prompt refinement, such as explicit no-claim-without-evidence instructions and post-generation claim verification.

##### 7.3.3.1 Key findings

Evidence-grounded explanations outperformed unconstrained explanations on all three evaluated dimensions. Structural groundedness was 90.0% for evidence-grounded explanations and 0.0% for unconstrained explanations. Under the stricter semantic-groundedness criterion, evidence-grounded explanations achieved 68.0% claim support, while unconstrained explanations achieved 0.0%. Usefulness was also higher for evidence-grounded explanations (3.74 ± 0.44 vs. 2.84 ± 0.37, mean ± SD). A Fisher exact test on strict semantic groundedness showed a significant difference between the two conditions (two-sided *p* = 1.70e−14). These results provide stronger evidence for explanation traceability and claim support than the earlier pilot subset, while still falling short of a standardized hallucination benchmark or classroom effectiveness study.

#### 7.3.4 Reproducibility check (multiple LLM runs)

As a small descriptive robustness check, we re-generated evidence-grounded explanations for 10 purposively selected instances with three independent runs per instance using DeepSeek V4 Flash (temperature = 0.7, max_tokens = 512). The sample was selected to cover successful, partially grounded, structurally weak, and truncated cases from the 50-instance evaluation pool; it is therefore not treated as a population-level significance test. Because this diagnostic used a higher temperature than the 100-output evaluation (0.7 vs. 0.3), its strict-semantic rate should not be directly compared with the 68.0% rate in Table 7 as if both were generated under identical conditions. As summarized in Table 8, structural groundedness was stable across all 30 outputs (30/30), indicating that citation-constrained prompting reliably induced explicit references to evidence elements in this selected subset. Strict semantic groundedness was lower and varied across instances (23/30, 76.7%; instance-level mean 0.77 ± 0.35), and 21/30 outputs still contained unsupported strategy, resource, or performance claims. In addition, 17/30 outputs appeared truncated under the 512-token cap. A truncation split showed that truncated outputs had lower usefulness (2.94 ± 0.56, *n* = 17) than non-truncated outputs (4.00 ± 0.00, *n* = 13), while unsupported claims were more frequent among non-truncated outputs (13/13) than truncated outputs (8/17). This pattern suggests a practical length-faithfulness trade-off: when the model has enough generation length to complete the explanation, it often adds study-strategy or performance-prediction claims beyond the evidence bundle; truncated outputs are less useful and, in some cases, stop before adding additional unsupported claims, although 8/17 truncated outputs still contained at least one unsupported claim. Overall usefulness averaged 3.40 ± 0.67 at the run level and 3.40 ± 0.49 at the instance level. These results support the traceability benefit of the prompting strategy while also clarifying that evidence grounding does not fully eliminate unsupported generalization or generation-length failures. Because this check uses a small purposive subset, a different temperature setting, and a single evaluator, we treat it as diagnostic evidence rather than a formal inferential reproducibility study.

**Table 8:** Multiple LLM run reproducibility check (10 sampled instances × 3 runs).

Sample	Structural groundedness	Strict semantic groundedness	Usefulness mean ± SD	Truncated	Unsupported claim
S001	3/3	0/3	3.33 ± 0.58	2/3	2/3
S005	3/3	3/3	4.00 ± 0.00	1/3	3/3
S008	3/3	3/3	3.33 ± 0.58	2/3	2/3
S012	3/3	2/3	3.67 ± 0.58	1/3	3/3
S020	3/3	2/3	4.00 ± 0.00	0/3	3/3
S021	3/3	3/3	3.33 ± 0.58	2/3	2/3
S033	3/3	3/3	3.67 ± 0.58	1/3	3/3
S039	3/3	3/3	3.33 ± 0.58	2/3	1/3
S043	3/3	3/3	3.00 ± 0.00	3/3	2/3
S047	3/3	1/3	2.33 ± 1.15	3/3	0/3
Overall	30/30	23/30	3.40 ± 0.67	17/30	21/30

## 8 Qualitative analysis: case studies on fabricated-history risk

To illustrate the effect of evidence-grounded prompting, we performed a comparative case study (Table 9), in which the same learner state and question were fed to both an unconstrained large language model and the proposed prompting framework.

**Table 9:** Qualitative contrast between unconstrained and evidence-grounded explanations.

Case	Input evidence	Unconstrained output risk	Evidence-grounded output property
Phantom history	No prior attempt record for target problem	Invents nonexistent assignments/attempts	Explicitly acknowledges missing history and cites evidence
Skill weakness	Low mastery score for target skill	May assert incorrect mastery narrative	References mastery numerically; ties advice to skill
Study strategy	Strategy suggestions	Can imply unsupported causes	Strategy remains generic unless supported; citations enforce provenance

**Figure 3:**
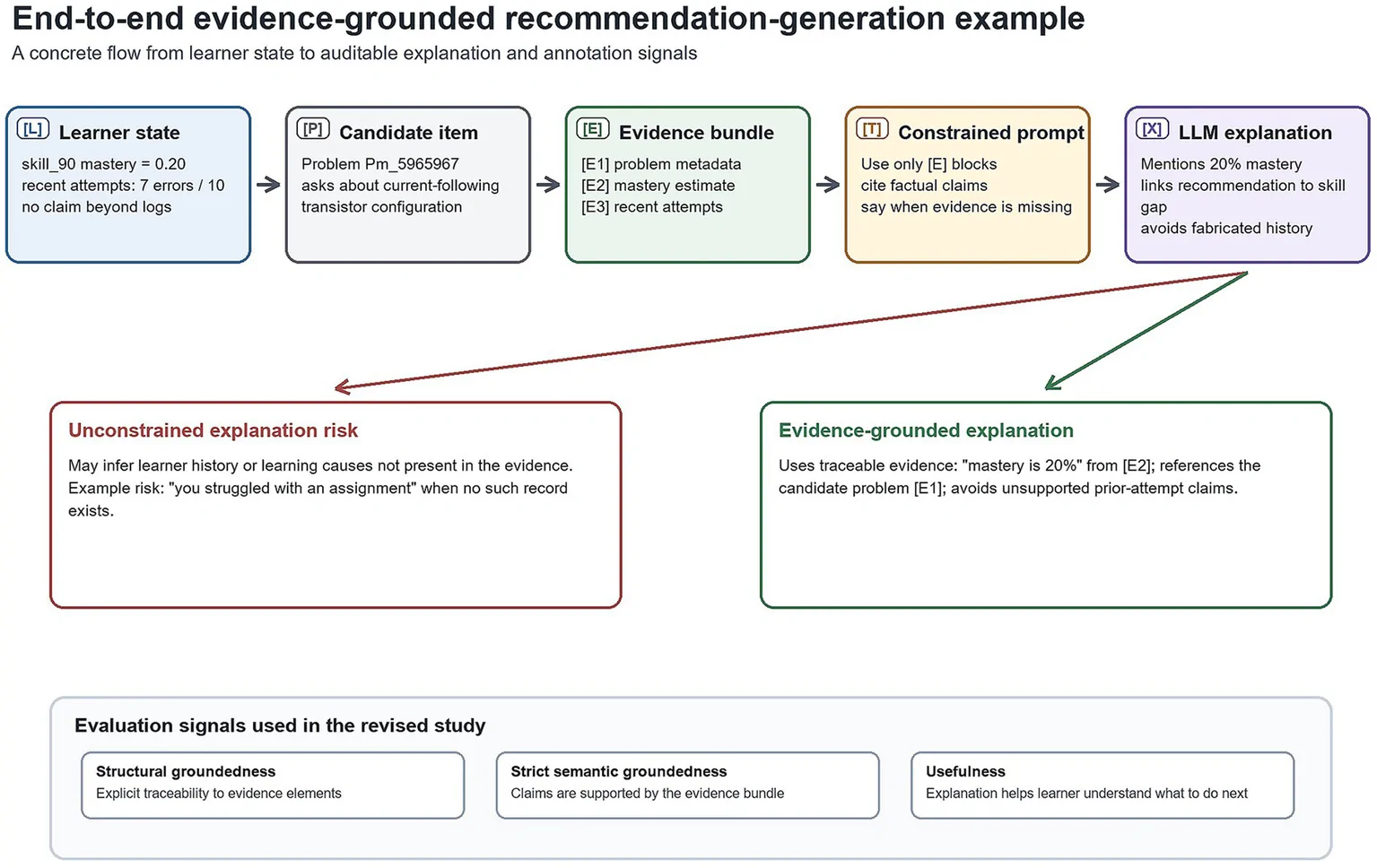
End-to-end recommendation-generation example. The figure illustrates how learner-state evidence, candidate-item metadata, and recent-attempt records are assembled into a structured evidence bundle, injected into a citation-constrained prompt, and converted into an auditable explanation. It also shows how the revised evaluation separates structural groundedness, strict semantic groundedness, and usefulness. The example illustrates the revised annotation boundary: structural citation is stricter than semantic claim support.

Figure 3 provides a concrete end-to-end example of how a learner state, a candidate problem, and a structured evidence bundle are converted into a citation-constrained explanation and then evaluated using structural groundedness, strict semantic groundedness, and usefulness.

### 8.1 Case 1: the “phantom” history (unconstrained)

Scenario: Learner has a weak mastery in “Recursion” but has never attempted “Problem 102.”

“I suggest that you do Problem 102 because you had some trouble with a prerequisite on Assignment 4. Reviewing your mistakes there will help you solve this problem more quickly.”

However, the model in this case invents an unsupported assignment history and creates a non-existent past interaction, which may undermine learner trust. This example illustrates the risk of unverified personalization in educational recommendations.

### 8.2 Case 2: evidence-grounded generation (ours)

Evidence Input (Simplified).

- [E2] Mastery: Recursion = 0.2.
- [E3] History: no attempts for Problem 102.

“This problem is recommended to strengthen your ‘Recursion’ skill, which is currently at a novice level (0.2) [#E2]. Since you have not tried this particular format before [#E3], start by identifying the base case and writing the recurrence step by step before coding.”

The evidence-grounded output identifies the learner’s skill gap and refers to the relevant mastery score. Although the explanation is less open-ended than the unconstrained output, it is traceable to the available evidence.

In contrast, unconstrained models can be perceived as more imaginative; but in the educational context, such creativity can become problematic if it leads to fictitious learner histories or incorrect claims in mathematics. The proposed constraints are designed to make the model acknowledge missing evidence rather than invent unsupported learner history, although this should be interpreted as risk reduction rather than a guarantee that all hallucinations are eliminated.

## 9 Discussion

### 9.1 Why evidence-grounded explanations improve human–AI trust

The advent of LLMs in education raises trust as an important practical challenge. Learners have to believe that the system’s guidance is aligned with their individual learning needs. Evidence-grounded explanations can help calibrate this trust: by citing sources, learners and educators can verify the rationale behind recommendations. Grounding is therefore not only a technique for reducing hallucination risk, but also a trust-building mechanism consistent with educational theory.

### 9.2 The “cold-start” efficiency dilemma

The simulation revealed that the attempt-count efficiency strategy (full) performed similarly to the gain-only strategy. This result suggests that attempt count is a delayed signal: early in learning, most learners have few attempts, so the efficiency term provides limited differentiation. As interaction histories grow, the signal can better distinguish fast progressors from learners who may need additional support.

To address this limitation, we use a hierarchical efficiency signal that also incorporates learning speed, approximated as the first-order change in mastery over recent attempts. After three to five observations, the system can estimate whether mastery is increasing quickly or slowly. Learners with positive learning velocity may receive higher efficiency scores, whereas learners with slower progress may receive lower scores. This design supports earlier differentiation while remaining interpretable. The ranking formula remains unchanged, but the static efficiency term eff(s) is replaced with a hierarchical calculation that activates the appropriate efficiency signal according to the available learner data.

### 9.3 What “LLM for recommendation” means here

We do not assert that LLMs directly improve ranking accuracy in this framework. Instead, the recommender first selects items through deterministic pedagogical signals, and the LLM is invoked only afterward to explain the selected item using labeled evidence. In this view, the LLM functions as a constrained natural-language interface for explanation and scaffolding. This differs from LLM-as-ranker systems, where the model participates in item selection, and from generic RAG systems, where retrieved text may serve a broad question-answering task rather than a learner-state-specific recommendation explanation.

## 10 Limitations

This work focuses on deployability and reliability, but several limitations should be noted.

The online mastery update is lightweight, interpretable, and stable, but it may not capture complex learning processes as well as more advanced knowledge-tracing methods.

Most importantly, the ranking framework has not yet been validated on a longitudinal real learner interaction dataset. The offline simulations rely on assumptions about learner behavior, response probability, and mastery growth, and the reported ranking differences may change when real-world learner logs are used. Future work should evaluate the framework with actual learner histories, delayed post-test outcomes, and classroom or platform-level deployment data.

The explanation evaluation is larger than the initial pilot but remains limited by its single-evaluator design. It supports a controlled comparison of traceability, strict claim support, and perceived usefulness across 100 generated outputs, but it does not replace multi-rater human evaluation or real learner interaction data. The multiple-run diagnostic uses a higher temperature than the 100-output evaluation (0.7 vs. 0.3), so its 76.7% strict-semantic rate should be interpreted as a separate robustness diagnostic rather than as a direct improvement over the 68.0% Table 7 result. Several low-scoring outputs also reflect generation failures such as truncation or missing evidence references, which are treated as part of the evaluated system behavior rather than removed from the analysis. Effective grounding also depends on accurate evidence metadata and relevance labels; noisy or misaligned evidence fields can affect strict semantic judgments. Future work should add independent annotators, agreement analysis, post-generation claim verification, and learner-facing usefulness ratings collected during real platform use.

Effective grounding depends on the completeness and quality of the evidence bundle; incomplete or noisy metadata may reduce explanation specificity and faithfulness.

LLM calls are made on demand, but inference cost and service availability remain practical limitations for large-scale deployment.

Deployment measurement. The same 30 successful DeepSeek V4 Flash calls provide a small empirical measurement of latency and token cost for this prompt setting. Mean latency was 8.605 s, median latency was 8.753 s, and the highest observed latency was 9.546 s. The calls used a mean of 617.9 prompt tokens and 474.6 completion tokens. Under the local pricing assumption used in our measurement script (1.0 CNY per 1M input tokens and 2.0 CNY per 1M output tokens), the estimated cost was approximately 0.001567 CNY per explanation. These values should be interpreted as measurements for the selected subset and sequential API setting, not as a full production throughput benchmark. Direct per-explanation API cost appears low, but response latency, prompt length, truncation under token limits, caching policy, timeout handling, concurrency, and provider availability remain important deployment constraints.

## 11 Conclusion

We propose an evidence-grounded closed-loop model for educational recommendation with generative explanations. In this system, deterministic recommendation is decoupled from generative explanation, and a citation-based grounding mechanism is used to make explanations more verifiable and to support calibrated trust. Simulation-based quantitative experiments demonstrate feasibility and closed-loop behavior under controlled assumptions, while the 100-output explanation evaluation shows that evidence-grounded prompting improves structural traceability, strict semantic support, and perceived usefulness relative to unconstrained generation. The current evidence remains preliminary for learning outcomes: real learner-log validation, larger multi-rater explanation evaluation, and deployment measurements are required before making strong claims about classroom effectiveness or production scalability.

## Data Availability

Publicly available datasets were analyzed in this study. This data can be found at: https://github.com/THU-KEG/MOOCCubeX.
